# Brief report: Circulating markers of fibrosis are associated with immune reconstitution status in HIV-infected men

**DOI:** 10.1371/journal.pone.0191606

**Published:** 2018-01-30

**Authors:** F. A. Tobolowsky, N. Wada, O. Martinez-Maza, L. Magpantay, S. L. Koletar, F. J. Palella, T. T. Brown, J. E. Lake

**Affiliations:** 1 Department of Internal Medicine, Division of Infectious Diseases, University of Colorado, Denver, Colorado, United States of America; 2 Department of Internal Medicine, Division of Infectious Diseases, University of Texas Health Science Center at Houston, Houston, Texas, United States of America; 3 Department of Epidemiology, Division of General Epidemiology and Methodology, Johns Hopkins University, Baltimore, Maryland, United States of America; 4 Department of Obstetrics and Gynecology, Epidemiology, Microbiology, Immunology & Molecular Genetics, University of California Los Angeles, Los Angeles, California, United States of America; 5 Department of Internal Medicine, Division of Infectious Diseases, Ohio State University, Columbus, Ohio, United States of America; 6 Department of Internal Medicine, Division of Infectious Diseases, Northwestern University, Chicago, Illinois, United States of America; 7 Department of Internal Medicine, Division of Endocrinology, Diabetes and Metabolism, Johns Hopkins University, Baltimore, Maryland, United States of America; 8 Department of Internal Medicine, Division of Infectious Diseases, University of California Los Angeles, Los Angeles, California, United States of America; University of Pittsburgh Centre for Vaccine Research, UNITED STATES

## Abstract

**Introduction:**

Lymphoid tissue fibrosis may contribute to incomplete immune reconstitution on antiretroviral therapy (ART) via local CD4^+^ T lymphocyte (CD4) depletion. Hyaluronic acid (HA) increases with fibrotic burden. CXCL4 concentrations increase in response to pro-fibrotic stimuli, but lower CXCL4 concentrations in HIV-infected individuals may reflect successful immune evasion by HIV. We investigated relationships between circulating HA and CXCL4 concentrations and immune reconstitution on ART in HIV-infected Multicenter AIDS Cohort Study participants.

**Methods:**

HIV-infected men on ART for >1 year with cryopreserved plasma samples and suppressed post-ART HIV-1 RNA were included. Men with post-ART CD4 <200 cells/mm^3^ were defined as immunologic non-responders (n = 25). Age-/race-matched men with post-ART CD4 >500 cells/mm^3^ served as controls (n = 49). HA and CXCL4 concentrations were measured via ELISA.

**Results:**

Median pre-ART CD4 was 297 cells/mm^3^ for non-responders vs 386 cells/mm^3^ for controls. Median post-ART CD4 was 141 cells/mm^3^ for non-responders and 815 cells/mm^3^ for controls. HIV infection duration was 23 years, with median time on ART 13 years for non-responders vs 11 years for controls. Pre-ART HA and CXCL4 concentrations did not vary by eventual immune reconstitution status. Post-ART HA concentrations tended to be higher (85 vs 36 ng/mL, p = 0.07) and CXCL4 concentrations were lower (563 vs 1459 ng/mL, p = 0.01) among non-responders. Among men with paired pre-/post-ART samples, non-responders had greater HA increases and CXCL4 decreases than controls (HA: 50 vs 12 ng/mL, p = 0.04; CXCL4: -1258 vs -405 ng/mL, p = 0.01).

**Conclusions:**

Higher circulating concentrations of HA and lower concentrations of CXCL4 are associated with failure of immune reconstitution on ART.

## Introduction

HIV infection is associated with chronic inflammation and immune activation that contributes to the development of end-organ disease [1]. When tissues are injured, the immune system initiates local inflammatory responses to promote wound healing via a transforming growth factor-β1 (TGF-β1)-mediated process [2–4]. In the presence of a chronic inflammatory stimulus, such as HIV infection, wound healing can become dysfunctional, resulting in excess extracellular matrix deposition and tissue fibrosis. Over time, fibrosis can lead to organ dysfunction.

Tissue fibrosis among HIV-infected persons may contribute to multiple non-communicable chronic diseases, including cardiovascular disease and neurocognitive decline [5,6]. Since non-AIDS events are now the leading cause of morbidity and mortality in persons on virally suppressive antiretroviral therapy (ART), clinical outcomes that may be associated with tissue fibrosis in HIV-infected persons warrant further exploration [7–9]. Additionally, lymphoid tissue fibrosis occurs early in HIV infection and contributes to CD4^+^ T lymphocyte depletion and immune system dysfunction [10, 11], a process that may not fully reverse following ART initiation [10].

Circulating biomarkers of tissue fibrosis include TGF-β1, hyaluronic acid (HA) and CXC chemokine ligand 4 (CXCL4) [12–15]. TGF-β1 is the canonical driver of fibrosis, but circulating concentrations can be challenging to measure [2,14,15]. TGF-β1 is secreted by activated platelets as they are recruited to sites of injury, as are T helper lymphocytes, macrophages, and mast cells during immune activation [16]. CXCL4, also known as platelet factor 4, is released from activated platelets and plasmacytoid dendritic cells. CXCL4 concentrations mirror TGF-β1 concentrations, and CXCL4 can reliably be measured in serum and plasma [17,18]. Additionally, *in vitro* data suggests that CXCL4 may interact with gp120 to prevent HIV-1 infection of CD4^+^ T lymphocytes [19,20]. HA is an extracellular matrix component produced during wound healing, and circulating concentrations are increased in fibrotic diseases [13,21–23]. In HIV infection, HA has been associated with a 4.6-fold increased risk of death or AIDS diagnosis, and an 8.7-fold increased risk of immune reconstitution inflammatory syndrome within twelve months of ART initiation [24].

In individuals who do not achieve substantial increases in CD4+ T lymphocyte counts following ART initiation, concentrations of circulating biomarkers of fibrosis and their relationships to CD4^+^ T lymphocyte recovery are not known. Given that incomplete immune reconstitution with ART initiation may be related to lymph node fibrosis, we hypothesized that circulating concentrations of fibrosis markers would be associated with immune reconstitution status. Therefore, we aimed to investigate relationships between concentrations of circulating fibrosis biomarkers pre- and post-ART initiation and immune reconstitution status in HIV-infected Multicenter AIDS Cohort Study (MACS) participants.

## Methods

The MACS is an ongoing, multicenter (Pittsburgh, PA; Baltimore, MD/Washington, DC; Chicago, IL; and Los Angeles, CA), prospective observational cohort study in which over 7,000 participants have been evaluated semi-annually by interviews, physical examination, and laboratory tests. The MACS was created in 1984 to study the natural history of AIDS among men who have sex with men, to identify risk factors for infection, and to obtain biological specimens for further study [25]. All participants provided written informed consent, and the study was approved by the University of California, Los Angeles IRB (reference number 10–001677 AM-00022).

We performed a case-control study of HIV-infected MACS men who had been on ART for ≥1 year and had HIV-1 RNA <500 copies/mL at the post-ART visit. All participants provided written informed consent, and the study was approved by the University of California, Los Angeles IRB (reference number 10–001677). Immunologic non-responders (cases) were defined as participants with a current absolute CD4^+^ T lymphocyte count <200 cells/μL after more than one year of ART (n = 25). Immunologic responders (controls) were defined by current absolute CD4^+^ T lymphocyte count ≥500 cells/μL (after more than one year of ART, n = 49), and were age (within 5 years)- and race-matched to cases. All participants had HIV-1 RNA <500 copies/mL at the post-ART visit.

Variables included for characterization of the population included: age (years), black race, current smoking, BMI (kg/m^2^), hepatitis B virus (HBV) infection (defined as positive HBV surface antigen or diagnosis of chronic HBV infection), hepatitis C virus (HCV) infection (defined by HCV RNA positivity), CD4^+^ T lymphocyte count (cells/mm^3^), HIV viral load (copies/mL), years since viral load <500, time since HIV diagnosis (years), time on ART (years), time on highly-active ART (years), current protease inhibitor (PI) use, current non-nucleoside reverse transcriptase inhibitor (NNRTI) use, prior zidovudine (AZT) or stavudine (D4T) use, and abacavir use since last visit. The variables were collected by self-report unless otherwise defined.

CXCL4 and HA concentrations were measured in pre- and post-ART plasma samples by ELISA (R&D Systems, Inc., Minneapolis, MN, USA) at the University of California, Los Angeles. To ensure standardization, internal lab quality control samples were run in duplicate on each plate. Additionally, two randomly chosen MACS samples per plate were run in duplicate. All other samples were run as singlets. For CXCL4, the mean within- and between-plate coefficients of variation were 4.1% and 6.1%, respectively. For HA, the mean within- and between-plate coefficients of variation were 4.1% and 3.6%, respectively.

Wilcoxon rank sum tests were used to compare HA and CXCL4 concentrations, and odds ratios were used to evaluate relationships between post-ART HA and CXCL4 concentrations to determine the likelihood of immune reconstitution. These exploratory analyses were hypothesis generating, and performed without adjustment for multiple testing. A two-sided α = 0.05 was used to determine statistical significance for this pilot study.

## Results

### Patient population

Overall, 74 participants were assessed at the post-ART visit, including both immunologic non-responders (n = 25) and controls (n = 49). However, only 43 participants (28 non-responders, 15 controls) had pre-ART samples, and only 27 participants had paired pre- and post-ART samples (non-responders n = 14, controls n = 13).

### Demographic and clinical characteristics at both the pre- and post-ART visit

Briefly, at the post-ART visit, the median age was 51 years for non-responders (n = 25) and 48 years for controls (n = 49). Thirty two percent of non-responders and 61% of controls self-reported black race. Statistically similar proportions of current smoking, proportions of HBV and HCV co-infection, and BMI distributions were observed among non-responders and controls, respectively. The median CD4^+^ T lymphocyte count was 141 cells/mm^3^ among non-responders and 815 cells/mm^3^ among controls. The median time since HIV diagnosis was 23 years for both groups. Time on ART was a median of 13 years for non-responders vs 11 years for controls. Sixty percent of non-responders were on PI-based ART vs 29% of controls, and 32% of non-responders were on NNRTI-based ART vs 59% of controls. Non-responders have more PI-based HAART (n = 15) with less NNRTI-based HAART (n = 8). Eighty-eight percent of non-responders and 73% of controls had prior AZT or D4T use.

At the pre-ART visit, the median age was 38 years for both immunologic non-responders (n = 28) and controls (n = 15). The median CD4^+^ T lymphocyte count was 297 cells/mm^3^ among non-responders and 386 cells/mm^3^ among controls. Median time since HIV diagnosis was 10 years for non-responders vs 13 years for controls.

### HA and CXCL4 concentrations pre- and post-ART

**Fig 1:**
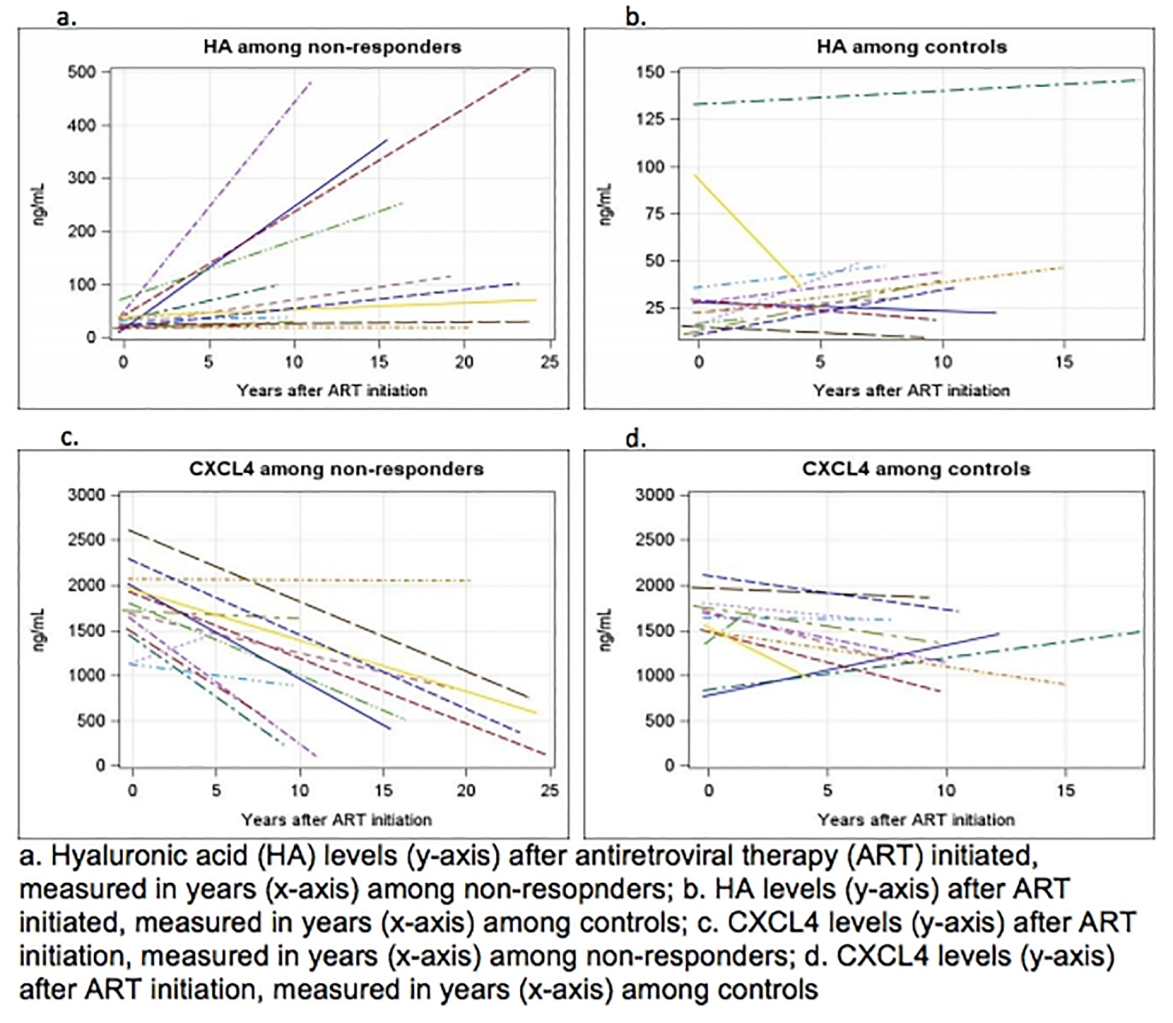
On-ART changes in HA and CXCL4 levels by immune reconstitution status.

At the pre-ART visit, concentrations of HA (non-responders 25 ng/mL vs 23 ng/mL controls, p = 0.44) and CXCL4 (non-responders 1760 ng/mL vs 1695 ng/mL controls, p = 0.73) did not vary depending on eventual immune reconstitution status. However, men with failure to immune reconstitute at the post-ART visit had higher median HA concentrations (non-responders 49 ng/mL vs 32 ng/mL controls, p = 0.004) and lower median CXCL4 concentrations (non-responders 858 ng/mL, 1601 ng/mL controls, p = 0.02). Overall, median increases in HA concentrations were greater for non-responders vs controls (50 ng/mL vs 12 ng/mL, p = 0.04), and median declines in CXCL4 concentrations were greater for non-responders vs controls (-1258 ng/mL vs -405 ng/mL, p = 0.01) (Fig 1). Additionally, changes in HA and CXCL4 were statistically significant only among non-responders (non-responders: HA p<0.001, CXCL4 p<0.001; responders: HA p = 0.13, CXCL4 p = 0.24). These changes in concentration were only measured in those who had paired pre- and post-ART samples.

## Discussion

In this case control study of HIV-infected MACS men, we observed that men who failed to immune reconstitute on ART had higher post-ART concentrations of HA and lower post-ART concentrations of CXCL4. Higher HA concentrations may correlate with greater lymphoid tissue fibrosis, which may lead to the inability to immune reconstitute on ART [13,26]. Given the association of CXCL4 with disease burden in non-infectious fibrotic diseases such as cystic fibrosis, higher CXCL4 concentrations were also anticipated among immunologic non-responders [17]. However, this expected relationship was not observed, and may, in fact, be reversed in HIV infection. In HIV-uninfected persons, CXCL4 concentrations can increase in pro-fibrotic states, inhibiting further TGF-β1 expression [12]. As such, recombinant CXCL4 therapy has been proposed for therapeutic use to improve immunologic function [27]. However, in viral infections, failure to produce CXCL4 may reflect a defective immunologic response to infection. For example, in mouse influenza models, lower concentrations of CXCL4 are associated with decreased viral clearance [28]. Similarly, higher concentrations of CXCL4 may prevent HIV-1 infection of T lymphocytes [19,20]. Thus, lower CXCL4 concentrations in immunologic non-responders may actually reflect greater immunologic devastation, lower capacity to control HIV infection and, ultimately, greater fibrosis burden.

It is important to note that even controls had high concentrations of HA post-ART (compared to HIV-uninfected persons, data not shown), consistent with the long durations of the time these men had lived with both treated and untreated HIV infection. The median duration of HIV infection among men in our cohort was 23 years. For controls and non-responders, respectively, the median time on ART was 11 and 13 years, and the median time on highly-active ART was 8 and 9 years. Thus, both immunologic responders and non-responders had a prolonged period without virologic suppression, which has become less common in the modern ART era. These data are also consistent with HIV infection as a chronic inflammatory stimulus. Indeed, in the “Berlin” patient, levels of lymphoid fibrosis similar to HIV-uninfected donors were observed following HIV cure [10], suggesting that removal of the pro-fibrotic stimulus allows for wound healing processes to occur.

This study has several limitations. First, as was common in the early ART era, many men experienced delayed ART initiation, persistent viral replication and continued circulating CD4^+^ T lymphocyte depletion even after ART initiation. For this reason, the results cannot be generalized to individuals initiating modern ART regimens and/or those who initiate ART shortly after HIV infection. Second, there is an imbalance between cases and controls in the median pre-ART CD4 T cell counts. Further, given sex differences in immune activation [29], these data are not necessarily reflective of HA and CXCL4 concentrations in HIV-infected women. Third, this study was performed retrospectively on banked samples and was therefore not randomized, allowing for clinical and demographic differences between the two groups. Fourth, although these biomarkers have been implicated in fibrosis in other disease states, it is still experimental in HIV. Lastly, our small sample size prevented better adjustment for measured variables that might function as confounders.

Despite these limitations, to our knowledge, this is the first study to document higher HA and lower CXCL4 concentrations in HIV-infected persons with persistent failure to immune reconstitute on ART, and provides *in vivo* evidence to support the hypothesis that the ability to produce CXCL4 may, in part, reflect a protective immunologic response against progression of HIV infection. These data also suggest that HA and CXCL4 have potential for future use both as non-invasive biomarkers of fibrosis in HIV clinical and research settings, and as targets for therapeutic interventions.

## Supporting information

S1 Table Demographic and clinical characteristics. *Median and interquartile range or percent; ^⌘^Subset of men with both pre-/post-ART samples have similar distributions; ^1^HBV surface antigen positive; ^2^HCV RNA positivity.
(TIFF)

S1 Text Minimal data set. (XLSX)
