# Supplementary figures and images for: Brief report: Circulating markers of fibrosis are associated with immune reconstitution status in HIV-infected men
Source: PLoS One. 2018 Jan 30;13(1):e0191606. doi: 10.1371/journal.pone.0191606 (PMC5790272; doi:10.1371/journal.pone.0191606)

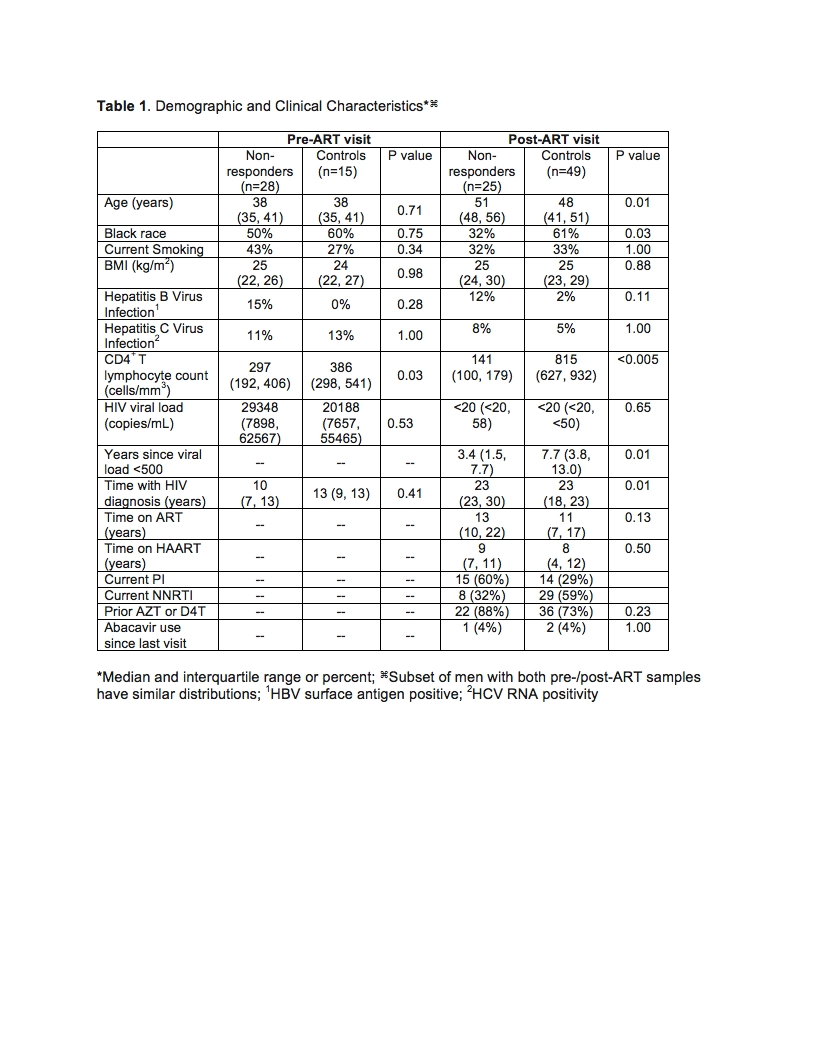

Supplement: S1 Table — *Median and interquartile range or percent; ⌘Subset of men with both pre-/post-ART samples have similar distributions; 1HBV surface antigen positive; 2HCV RNA positivity. (TIFF) [file pone.0191606.s001.tiff]
